# Retinal thickness measurements in sickle cell patients with HbSS and HbSC genotype

**DOI:** 10.1016/j.jcjo.2017.10.006

**Published:** 2018-08

**Authors:** Wei S. Lim, Tejal Magan, Omar A. Mahroo, Pirro G. Hysi, Juliana Helou, Moin D. Mohamed

**Affiliations:** * Ophthalmology Department, St Thomas’ Hospital, London, United Kingdom; † NIHR Biomedical Research Centre at Moorfields Eye Hospital NHS Foundation Trust and the UCL Institute of Ophthalmology, London; ‡ Section of Academic Ophthalmology, School of Life Course Sciences^,^ Faculty of Life Course Sciences and Medicine, King’s College London, St Thomas’ Hospital Campus, London, United Kingdom

## Abstract

**Objective:**

Temporal macula thinning has been reported in sickle cell patients, but it remains unclear if there is a difference between HbSS and HbSC genotypes. We aimed to quantitatively compare macular thickness between eyes with HbSS and HbSC genotype.

**Design:**

Retrospective descriptive study.

**Methods:**

Consecutive patients seen over a 5.5-year period in the Ophthalmology Department at St Thomas’ Hospital, London, were identified. Macular optical coherence tomography images were retrospectively analyzed. The retinal thickness in all 9 subfields of the Early Treatment Diabetic Retinopathy Study (ETDRS) grid was compared between HbSS and HbSC eyes. Right eyes and left eyes were analyzed independently, as well as averaged measurements from both eyes. Comparison was made between the 2 genotypes, adjusting for age and sex, and for multiple testing. Scans were excluded in cases of poor fixation or ocular comorbidity affecting retinal thickness.

**Results:**

132 HbSC and 120 HbSS patients were identified. Scans from 166 right and 153 left eyes were included (with approximately equal numbers of HbSS and HbSC genotypes). Mean retinal thickness was lower in HbSS eyes compared with HbSC eyes in all subfields of the ETDRS grid, but in most subfields the difference was <10 microns. Differences reached statistical significance for outer superior, inferior, and temporal subfields and the inner temporal subfield (*p* < 0.05).

**Conclusion:**

Although the HbSC genotype is more strongly associated with proliferative retinopathy, HbSS patients had on average more macular thinning.

## RÉSUMÉ

**Objectif:**

On a observé un amincissement de la macula du côté temporal en présence d’anémie falciforme, mais on ne sait pas encore avec certitude s’il existe une différence entre les génotypes HbSS et HbSC. Nous avons entrepris de quantifier et de comparer l’épaisseur de la macula dans des yeux de génotype HbSS et de génotype HbSC.

**Nature:**

Étude descriptive rétrospective.

**Méthodes:**

Des patients consécutifs examinés sur une période de 5,5 ans au service d’ophtalmologie du *St Thomas’ Hospital*, London, ont été recensés. On a procédé à une analyse rétrospective des images de la macula obtenues par tomographie par cohérence optique. L’épaisseur rétinienne dans l’ensemble des 9 sous-champs de l’échelle ETDRS (*Early Treatment Diabetic Retinopathy Study*) a été comparée dans les 2 groupes d’yeux (HbSS et HbSC). Les yeux droits et les yeux gauches ont été analysés de manière indépendante, au même titre que les mesures moyennes issues des 2 yeux. On a comparé les 2 génotypes, après ajustement pour tenir compte de l’âge, du sexe et des évaluations multiples. Les images qui présentaient une mauvaise fixation ou faisaient ressortir une atteinte oculaire concomitante influant sur l’épaisseur rétinienne ont été rejetées.

**Résultats:**

Cent trente-deux patients de génotype HbSC et 120 de génotype HbSS ont ainsi été identifiés. Les images de 166 yeux droits et de 153 yeux gauches ont été admises à l’étude (représentation à peu près égale des génotypes HbSS et HbSC). L’épaisseur rétinienne moyenne était inférieure dans les yeux HbSS comparativement aux yeux HbSC pour tous les sous-champs de l’échelle ETDRS, bien que, dans la plupart des sous-champs, la différence ait été < 10 microns. Les différences atteignaient le seuil de signification statistique pour les sous-champs supérieur externe, inférieur et temporal, de même que pour le sous-champ temporal interne (*p* < 0,05).

**Conclusion:**

Bien que le génotype HbSC soit plus fortement associé à une rétinopathie proliférante, les patients porteurs du génotype HbSS avaient, en moyenne, plus d’amincissement maculaire.

Sickle cell disease is caused by abnormal hemoglobin in red blood cells causing red blood cells to assume a rigid sickle shape under certain circumstances, such as low oxygen concentration. The sickle-shaped blood cells can block capillaries, causing ischemia and end organ damage.^1^

The 2 main variants of sickle cell disease are HbSS and HbSC, determined by the mutated allele present. HbSS is caused by the presence of 2 copies of the mutated hemoglobin S (HbS) allele. The mutation results in a glutamic acid being substituted by valine at position 6 on the beta-globin chain.^2^ The mutation causes instability of the hemoglobin S molecules, causing red cells to assume a sickle-shaped conformation rather than a normal biconcave disc when metabolically stressed.

In the other major variant, HbSC, there is 1 HbS allele and another hemoglobin C (HbC) allele. The HbC allele is caused by substitution of a glutamic acid residue to a lysine residue at position 6 on the beta-globin chain. The HbC molecule is more stable than HbS; therefore, the HbSC genotype does not cause as much sickling as the HbSS genotype. As a result, HbSC patients experience fewer systemic vaso-occlusive events. However, it is well established that sickle cell patients with the HbSC genotype have a greater prevalence of proliferative retinopathy compared with sickle cell patients of HbSS genotype.^3^

Previous reports have shown that macular thinning is a common finding in sickle cell patients.^4^, ^5^ Our study was undertaken to establish whether there is a difference in macular thickness between sickle cell patients with HbSS and HbSC genotypes. Mathew et al.^6^ reported that macular thinning was associated more with HbSS compared with HbSC eyes but were cautious in making any conclusions because of an unequal distribution of the 2 genotypes in their cohort. Their results were based on qualitative analysis of the presence of temporal thinning, whereas in our study, we used quantitative retinal thickness measurements, obtained with spectral domain optical coherence tomography (OCT).

## Material and methods

Consecutive HbSS and HbSC patients seen in the retinal service at St Thomas’ Hospital, London, over a 5.5 year period were identified from the electronic patient record. Their macular OCT images were retrospectively reviewed. Scans that were poorly centered or from patients with other ocular comorbidities that could affect retinal thickness (such as an epiretinal membrane or diabetic maculopathy) were excluded. The most recent eligible scans were included.

**Fig. 1:**
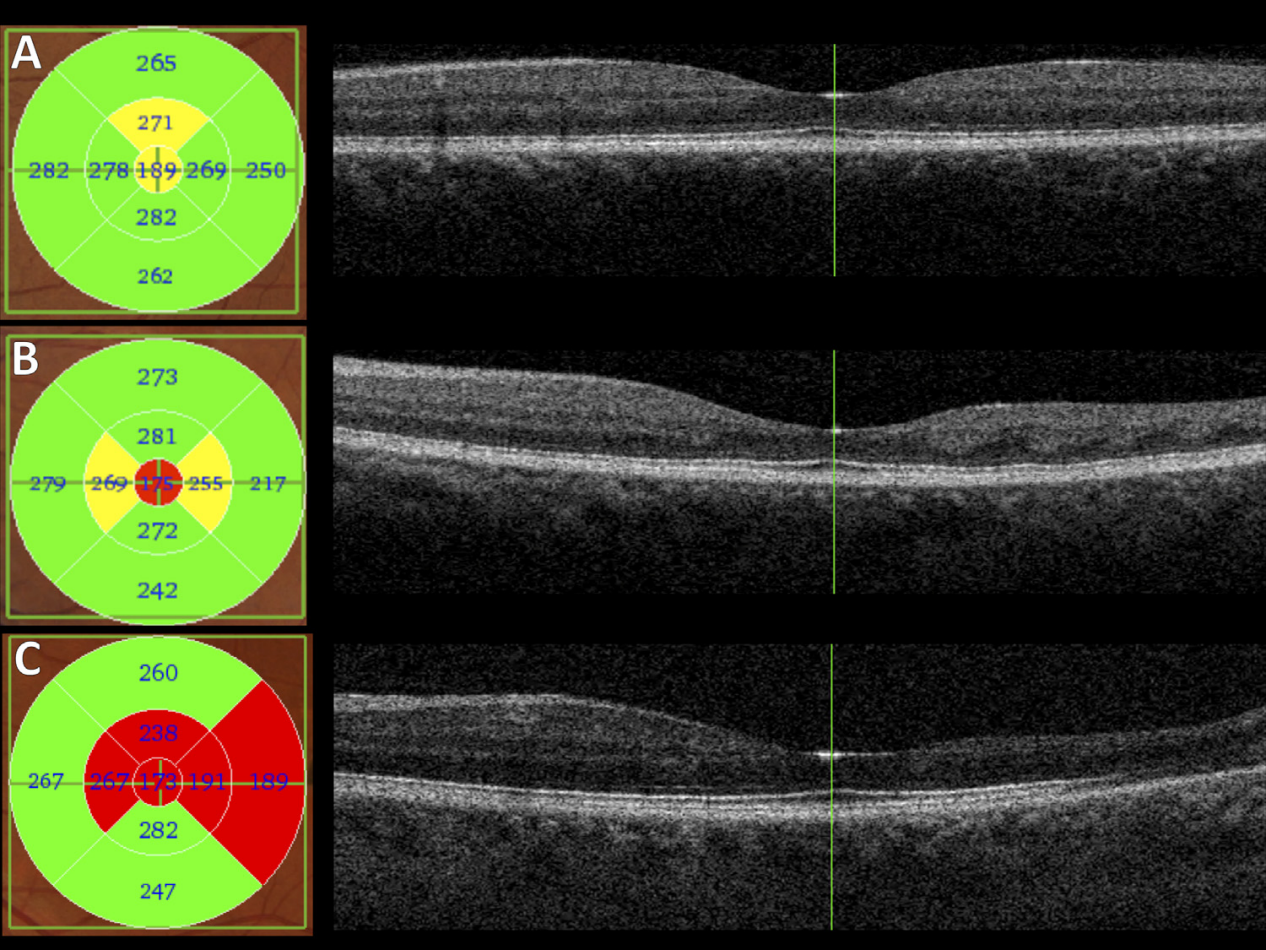
Examples of macular subfield thicknesses and OCT scans from 3 patients. A, Subfield thicknesses (left panel) and horizontal OCT scan through the foveal center (right panel) for an HbSC patient with minimal or no macular thinning. B, Corresponding images from a patient with HbSS genotype with mild temporal thinning. C, Images from another HbSS patient with more marked temporal thinning.

Macular OCT scans were obtained using a Topcon 3D OCT-2000 machine or 3D-OCT 1000 (Topcon Medical Systems, Oakland, N.J.); standard three-dimensional macula scans, 6.0 × 6.0 mm wide and 512 × 128 resolution, were analysed. The retinal thickness in all 9 sectors of the Early Treatment Diabetic Retinopathy Study (ETDRS) grid was recorded from the Topcon OCT viewing software (Fig. 1), after establishing that there were no segmentation errors. Measurements from the left and right eyes were then analyzed independently. Each reading was taken by 1 of 3 doctors involved in the data acquisition in this study.

The mean retinal thickness in each sector of the ETDRS grid was calculated for HbSS and HbSC eyes. The values obtained for right eyes and left eyes were initially analyzed separately, and then averaged data from both eyes were included per patient (unless only 1 eye was eligible). Thicknesses were compared between the HbSC and HbSS groups (unpaired *t* test). The results were then adjusted for age and sex (by performing a regression on age and sex and using the residuals for the *t* test). Those *p* values that emerged as significant (*p* < 0.05) were then corrected for multiple testing by using a permutation procedure (label-swapping with empirical *p* values compared with *p* values from 1000 randomly permuted *t* tests).

This study was retrospective and was conducted by the clinical care team, with subsequent analysis of anonymized data. Formal ethical approval was not required, as confirmed by the Guys & St Thomas’ Hospital Research & Development Department.

## Results

132 HbSC and 120 HbSS patients were identified. The right eyes of 35 HbSC patients and 30 HbSS were excluded because a macula OCT was unavailable or because of other retinal comorbidities that could affect macular thickness. An additional 14 right eyes of HbSC patients and 7 right eyes of HbSS patients were excluded because the scan, and therefore the ETDRS grid, was not adequately centered on the fovea. In total, the right eyes of 83 HbSC and 83 HbSS patients were included in the analysis. Proportions of right eyes excluded were similar for both genotypes (*p* = 0.29).

The left eyes of 39 HbSC patients and 27 HbSS patients were excluded because a macula OCT was unavailable or because of other retinal comorbidities that could affect macular thickness. An additional 19 left eyes of HbSC patients and 14 left eyes of HbSS patients were excluded because the ETDRS grid was not centered on the fovea. Hence, the left eyes of 74 HbSC and 79 HbSS patients were included in the analysis. Proportions of left eyes excluded were similar for both genotypes (*p* = 0.11).

Patient demographics are shown in Table 1. Sex distributions were similar for the 2 genotypes, but HbSS patients were significantly younger (*p* = 0.03).

**Table 1:** Demographics of HbSC and HbSS patients

	Right Eyes		Left Eyes	
	HbSC (n = 83)	HbSS (n = 83)	HbSC (n = 74)	HbSS (n = 79)
Mean age ± SD (years)	41 ± 12	37 ± 11	42 ± 12	37 ± 11
Sex, n (%)				
Female	58 (70)	57 (69)	50 (68)	55 (70)
Male	25 (30)	26 (31)	24 (32)	24 (30)

Figure 1 shows examples of macular OCT imaging from a patient with HbSC genotype and 2 patients with HbSS genotype.

Table 2 compares the macular thickness in all 9 subfields of the ETDRS grid for HbSS and HbSC patients. The right eyes and left eyes were analyzed separately.

**Table 2:** Macular thickness of HbSC and HbSS eyes in all subfields of the ETDRS grid

Subfield on ETDRS grid	Right Eyes			Left Eyes		
	HbSC (83 Right Eyes)	HbSS (83 Right Eyes)	*p* Value for Difference	HbSC (74 Left Eyes)	HbSS (79 Left Eyes)	*p* Value for Difference
	Mean in microns (SD)	Mean in microns (SD)		Mean in microns (SD)	Mean in microns (SD)	
Central	207 (24)	202 (20)	0.175	206 (22)	202 (21)	0.200
Inner superior	289 (24)	283 (29)	0.131	288 (22)	283 (31)	0.297
Inner nasal	289 (20)	285 (25)	0.322	290 (20)	288 (23)	0.562
Inner inferior	286 (26)	281 (31)	0.290	287 (21)	283 (26)	0.229
Inner temporal	275 (23)	266 (33)	0.039*	271 (21)	264 (38)	0.124
Outer superior	263 (19)	256 (18)	0.018*	263 (18)	259 (24)	0.329
Outer nasal	276 (19)	271 (20)	0.092	274 (20)	272 (22)	0.473
Outer inferior	256 (22)	250 (18)	0.075	255 (17)	250 (20)	0.107
Outer temporal	240 (24)	228 (26)	0.005*	241 (19)	233 (26)	0.022*

ETDRS, Early Treatment Diabetic Retinopathy Study.

Right and left eye measurements were not combined.

* Statistically significant difference between the 2 genotypes (*p* < 0.05).

Table 3 compares the macular thickness in all 9 subfields of the ETDRS grid for HbSS and HbSC patients, with the average of right and left eye measurements taken for each patient if both were available. Right-hand columns give raw *p* values (unpaired *t* test) and after adjustment for age and sex. Those *p* values found to be significant after the latter adjustment were corrected for multiple testing in the final column.

**Table 3:** Comparison of retinal thickness measurements between HbSS and HbSC genotypes with measurements averaged from both eyes (unless only 1 eye eligible for inclusion)

	Mean (SD) Retinal Thickness (microns)		*p* Values for Difference		
ETDRS subfield	HbSC (n = 91)	HbSS (n = 93)	Uncorrected	Adjusted for Age and Sex	With Multiple Testing Correction
Central	206 (23)	203 (20)	0.258	0.154	NA
Inner superior	288 (22)	284 (28)	0.255	0.045*	0.052
Inner nasal	289 (19)	287 (23)	0.570	0.193	NA
Inner inferior	286 (22)	282 (24)	0.305	0.086	NA
Inner temporal	272 (22)	266 (32)	0.088	0.024*	0.031*
Outer superior	262 (17)	258 (20)	0.145	0.027*	0.033*
Outer nasal	274 (19)	271 (20)	0.297	0.070	NA
Outer inferior	255 (20)	250 (17)	0.089	0.023*	0.029*
Outer temporal	240 (21)	230 (24)	0.005*	0.002*	0.004*

ETDRS, Early Treatment Diabetic Retinopathy Study; NA, not applicable.

*p* values are from unpaired *t* test. The correction for multiple testing was only applied to parameters with significant *p* values (after adjusting for age and sex).

* *p* < 0.05 was taken as significant.

The results show that the average retinal thickness in all subfields of the ETDRS grid were lower in HbSS eyes compared with HbSC eyes. The differences, although small, were statistically significant in the outer temporal subfield in both right and left eyes. After averaging data from both eyes, correcting for age, sex, and multiple testing, the differences were significant for all outer subfields (other than the nasal) and for the inner temporal subfield.

## Discussion

In this study, we retrospectively reviewed the macular OCT scans of a large consecutive cohort of patients with sickle cell disease and found that although central macular thickness measurements are similar in both HbSS and HbSC genotypes, HbSS patients had on average greater macular thinning for all macular subfields. This reached statistical significance in the superior, inferior, and temporal outer subfields and the inner temporal subfield.

These results are consistent with the previously suggested idea that HbSS patients are more likely to have macular thinning,^6^ particularly temporally, compared with those with HbSC. Our findings are consistent with different patterns of sickle cell retinopathy in HbSS and HbSC patients. Inner retinal loss in individuals with sickle cell disease has previously been explained by retinal ischemia caused by occlusion of the retinal vasculature.^7^, ^8^ Emerging data from OCT angiographic studies confirm the poor perifoveal circulation and suggest that fluorescein angiography may not be as sensitive in detecting poor perfusion.^9^ HbSS patients are known to have more systemic vaso-occlusive events, but they paradoxically have a milder phenotype in the peripheral retina from proliferative sickle retinopathy. Although HbSC patients have greater risk of proliferative retinopathy, in a recent study, HbSC genotype was not found to be a risk factor for visual loss. This may be explained in part by HbSS patients having more macular pathology contributing to sight loss, which would be supported by the findings of the present study.^10^ Using OCT angiography, Han et al. found lower density of deep capillary plexus at the temporal macula in HBSC eyes but no statistical difference in mean foveal nonflow area between HBSS and HBSC eyes.^11^

The pattern of subfield thinning is intriguing. Vessel occlusion leading to macular thinning has been shown to mainly affect the region temporal to the fovea as the temporal macular vasculature ends in the horizontal raphe acting like terminal vessels.^12^ The subfields corresponding to nonsignificant *p* values in Table 3 might show significant differences with a larger sample. If the 3-micron difference in central subfields were significant, approximately 700 or more patients would be required in each group to detect this with 80% power (although fewer individuals may be needed if the cohorts were age-matched or parameters age-adjusted). However, it is debatable whether such a small difference would be of clinical significance.

The macular thinning in HbSS has been associated with reduced retinal sensitivity, albeit with preserved central Snellen visual acuity.^13^ The disparity between the macula and the peripheral retina, with possibly more pronounced macular changes in HbSS, yet more peripheral proliferative changes in HbSC, remains difficult to explain. Hannemann et al. showed that HBSC red cells sickle via a slightly different mechanism from HBSS red cells involving different ion channels causing red cell dehydration.^14^ Different metabolic conditions in the peripheral retina and differences in vascular architecture with rates of blood flow might cause differential mechanisms, or effects, of sickling in the 2 genotypes. It is also possible that retinal thinning is still more pronounced in HbSS patients in the periphery, and the mechanism for development of proliferative retinopathy may not associate strongly with retinal thinning.

Some limitations of the present study deserve mention. The study was retrospective, and not all patients had available scans. The cohort of patients was those seen in a retina clinic, and it is possible that those with visual impairment would be overrepresented. However, because patients are referred routinely from hematology departments for annual eye screening, the majority were asymptomatic. Also, the mean age was different in the 2 cohorts: the HbSS patients were younger. However, retinal thickness (in noncentral subfields) declines with age,^15^ so the finding of reduced thickness in the HbSS patients would not appear to be attributable simply to the age difference because it is in the opposite direction; indeed, the *p* values became more significant after correction for age. We had to exclude a large number of eyes because of other pathologies that could affect macular thickness, such as epiretinal membranes, cystoid macular edema, drusen, macular hole, and previous retinal detachment surgery.

In conclusion, in this study we present data suggesting that on average HbSS patients had greater macular thinning than HbSC patients, especially in the outer macular subfields. It would also be interesting to look at retinal thickness of the peripheral retina outside the ETDRS grid, and emerging wider field OCT technologies may help answer the many remaining questions.

## Disclosure

Omar A. Mahroo has received funding from Fight for Sight UK, Birdshot Uveitis Society, Thomas Pocklington Trust, the National Institute of Health Research Biomedical Centre at Moorfields Eye Hospital and the UCL Institute of Ophthalmology, and the Wellcome Trust. The authors have no proprietary or commercial interest in any materials discussed in this article.
